# Assessment of Pediatric Telemedicine Using Remote Physical Examinations With a Mobile Medical Device: A Nonrandomized Controlled Trial

**DOI:** 10.1001/jamanetworkopen.2022.52570

**Published:** 2023-02-02

**Authors:** Rafaela Wagner, Thalita Cecília Lima, Marielen Ribeiro Tavares da Silva, Anna Clara Pereira Rabha, Marinei Campos Ricieri, Mariana Millan Fachi, Rogério Carballo Afonso, Fábio Araújo Motta

**Affiliations:** 1 Department of Telehealth, Hospital Pequeno Príncipe, Curitiba, Paraná, Brazil; 2 Department of Clinical Research, Hospital Pequeno Príncipe, Curitiba, Paraná, Brazil; 3 Emergency Department, Sabará Hospital Infantil, São Paulo, Brazil; 4 Value Management Office, Hospital Pequeno Príncipe, Curitiba, Paraná, Brazil; 5 Department of Innovation and Business Development, Sabará Hospital Infantil, São Paulo, Brazil

## Abstract

**Importance:**

The number of innovations in health care based on the use of platforms, digital devices, apps, and artificial intelligence has grown exponentially in recent years. When used correctly, these technologies allow inequities in access to health care to be addressed by optimizing care and reducing social and geographic barriers. However, most of the technological health care solutions proposed have not undergone rigorous clinical studies.

**Objective:**

To assess the concordance between measurements from a remote physical examination using a mobile medical device and measurements from a conventional in-person physical examination.

**Design, Setting, and Participants:**

This nonrandomized controlled trial was conducted from January 1 to December 31, 2020. The clinical parameters compared were heart rate; body temperature; heart, lung, and abdominal auscultation; otoscopy; throat and oral examination; and skin examination. A total of 690 patients with clinical stability and various symptoms who were seen in the emergency department of 2 Brazilian pediatric hospitals were eligible to enter this study.

**Main Outcomes and Measures:**

The primary outcome was concordance between measurements from a telemedicine physical examination using a mobile medical device and measurements from a conventional in-person physical examination. The secondary outcome was the specificity and sensitivity of the digital device, considering the conventional in-person consultation as the gold standard.

**Results:**

Among 690 patients, the median (IQR) age at study entry was 5 (1-9) years; 348 (50.4%) were female, and 331 (48.0%) presented with a chronic disease. Regarding the primary outcome, the concordance values were 90% or greater for skin examination (94% for rash, 100% for hemorrhagic suffusion, and 95% for signs of secondary infection), characteristics of the mucosa (98% for hydration and 97% for coloring), and heart (95% for murmur, 97% for rhythms, and 98% for sounds), lung (91% for adventitious sounds, 97% for vesicular sounds, and 90% for fever), and abdominal (92% for abdominal sounds) auscultations. Concordance values were lower for otoscopy (72% for the ear canal and 86% for the tympanic membrane), throat and oral examination (72%), and rhinoscopy (79% for mucosa and 81% for secretion). The specificity was greater than 70% (ranging from 74.5% for the ear canal to 99.7% for hemorrhagic suffusion) for all variables. The sensitivity was greater than 52% for skin examination (58.0% for rash and 54.8% for signs of secondary infection), throat and oral examination (52.7%), and otoscopy (66.1% for the ear canal and 64.4% for the tympanic membrane).

**Conclusions and Relevance:**

In this study, measurements from remote physical examination with a mobile medical device had satisfactory concordance with measurements from in-person physical examination for otoscopy, throat and oral examination, skin examination, and heart and lung auscultation, with limitations regarding heart and lung auscultation in infants and abdominal auscultation in children of all ages. Measurements from remote physical examination via a mobile medical device were comparable with those from in-person physical examination in children older than 2 years. These findings suggest that telemedicine may be an alternative to in-person examination in certain contexts and may help to optimize access to health care services and reduce social and geographic barriers.

**Trial Registration:**

Brazilian Registry of Clinical Trials Identifier: RBR-346ymn

## Key Points

**Question:**

Are measurements from remote physical examination with a mobile medical device comparable with those from in-person examination in children?

**Findings:**

In this nonrandomized controlled trial of 690 pediatric patients, measurements from remote physical examination (via teleinterconsultation) with a mobile medical device were comparable with those from in-person physical examination in children older than 2 years, with satisfactory concordance for otoscopy, throat and oral examination, skin examination, and lung and heart auscultations.

**Meaning:**

These findings suggest that telemedicine may be an alternative to in-person examination in specific contexts, thereby optimizing access to health care services and reducing social and geographic barriers.

## Teaser

This nonrandomized controlled trial assesses the concordance between measurements from a telemedicine physical examination using a mobile medical device and measurements from a conventional in-person physical examination among pediatric patients.

## Introduction

Despite technological advances in clinical diagnosis, physical examination has substantial benefits for the prevention, diagnosis, and treatment of diseases. In the context of telehealth, especially in pediatric populations, a high-quality tool for collecting physical examination variables by means of portable medical devices could be a boon for telemedicine. The provision of patient health care outside of traditional health care settings has become increasingly common and gained importance during the COVID-19 pandemic, which highlighted the emerging need for technologies that can effectively and safely support remote consultations.

Various studies^1,2,3,4,5,6,7^ have compared the accuracy of digital devices with that of conventional methods (especially for heart auscultation and otoscopy). According to most of these studies, there was no statistically significant difference between conventional and digital devices, and the latter were sometimes superior.^1,2,3,4,5,6,7^

However, traditional telemedicine and remote monitoring in a pediatric setting involve specific challenges because patients of certain age groups and stages of development have a limited ability to express themselves and verbalize their feelings. In a study performed in Israel,^8^ a limitation reported by physicians who performed teleconsultations for acute symptoms was a feeling of insecurity because an adequate risk assessment was not being made; this feeling of uncertainty was primarily associated with the fact that a physical examination could not be performed in nonverbal patients.

Although difficulty in performing a complete physical examination is inherent to telemedicine, devices that allow data from a physical examination to be captured remotely, such as digital stethoscopes or otoscopes, are being increasingly used with the aim of minimizing this difficulty. As reported in previous studies,^5,9,10^ an easy-to-use mobile device (TytoPro; TytoCare) that functions as an otoscope and stethoscope has been developed; this device allows for the remote examination of the ears, throat, skin, heart, and lungs in a single device. Thus, the device allows the synchronous and asynchronous transmission of physical examination data from caregivers or health care professionals to the physician, enabling the removal of an important barrier to the implementation of telemedicine in specific contexts.^11^

Nevertheless, the majority of innovative technological health care solutions proposed have not undergone rigorous clinical studies, which are widely used for other products in the health care system. Consequently, many such solutions are implemented without clinical trials that can accurately estimate their effectiveness and safety when used in clinical practice settings, and some are even implemented without adequate management of postimplementation events.^12^ Therefore, the present study sought to assess the concordance between measurements from a telemedicine physical examination using a mobile medical device to capture data vs measurements from a conventional in-person physical examination.

## Methods

This prospective multicenter single-arm nonrandomized controlled trial was conducted to assess the ability of a mobile device to accurately perform remote physical examinations as part of a telemedicine consultation. The study compared a traditional physical examination with a teleinterconsultation (an exchange of audio and video information between physicians to aid diagnosis and treatment) using a mobile device in the emergency department of 2 Brazilian pediatric hospitals. The study was assessed and approved by the institutional review boards of Pequeno Príncipe Hospital and Sabará Hospital. The full trial protocol and statistical analysis plan are provided in Supplement 1. All patients and/or their legal representatives signed an informed consent form and an informed assent form that was adapted for the participant’s age. This study was conducted according to the Transparent Reporting of Evaluations with Nonrandomized Designs (TREND) guideline,^13^ and results were reported according to the Consolidated Standards of Reporting Trials (CONSORT) guideline.^14^

The medical mobile device used in this study (TytoPro) was designed to perform a physical examination during a teleconsultation and allows the remote evaluation of ears, throat, skin, heart, and lungs in a single device, as reported in previous studies.^5,9,10^ It consists of a touch-sensitive screen with an integrated high-resolution camera and infrared forehead thermometer to which a digital stethoscope, otoscope, or tongue depressor can be coupled. The device is paired with a mobile phone or tablet to allow videoconferencing while capturing data from a physical examination. The data can be transmitted synchronously or asynchronously. The physician accesses the information from a dashboard that also provides the patient’s details and information obtained during the consultation.^11^ The device as well as its dashboard and use of medical records comply with a series of national and international standards for data transmission and security.^10,11^ It is approved by the US Food and Drug Administration and complies with the Health Insurance Portability and Accountability Act.^15^ The device also complies with the General Data Protection Regulation of the European Union^16^ and is approved by the Agência Nacional de Vigilância Sanitária (the Brazilian Health Regulatory Agency) and the National Institute of Metrology, Standardization, and Industrial Quality for use in Brazil, where the study was conducted. The device was used to measure the following clinical parameters: heart rate; body temperature; heart, lung, and abdominal auscultation; otoscopy; throat and oral examination; and skin examination.

A total of 690 pediatric patients seen in the emergency department of 2 Brazilian pediatric hospitals between January 1 and December 31, 2020, were included (eFigure in Supplement 2). Eligible patients had stable conditions and low-complexity (nonurgent) symptoms (corresponding to green classifications indicating standard status on the adapted Manchester Triage System scale^17^). Sample size was calculated based on 2 statistical factors: comparison of paired proportions and measurement concordance between methods (examination using a mobile device vs conventional in-person examination) (more details are available in the trial protocol and statistical analysis plan in Supplement 1).

Patients were examined in both a synchronous teleinterconsultation and a conventional in-person consultation. After patients had been screened by a nurse and classified as having nonurgent symptoms, they were first assessed by a first-year pediatric resident using the device and a pediatrician located in a remote room who guided the resident through the teleinterconsultation. As the resident performed the examination, the data were viewed in real time and recorded by the pediatrician in the device’s dashboard, then transferred to the patient’s electronic health record using a data collection sheet specifically designed for the study. All physicians who participated in the study were trained to perform data collection. Because all of the data from the examinations were required to be collected, data loss was minimized.

The patient was then examined in person by a different pediatrician. The physician performed the complete routine procedure (medical history and physical examination) and completed the same data collection sheet. The traditional physical examination involved assessment of the same clinical parameters using standard equipment or procedures, such as a digital thermometer, conventional stethoscope, and otoscope. After this stage, a diagnosis or clinical management recommendation was proposed based on the findings of the second consultation. The flowchart of patient consultations is shown in the Figure.

**Figure.**
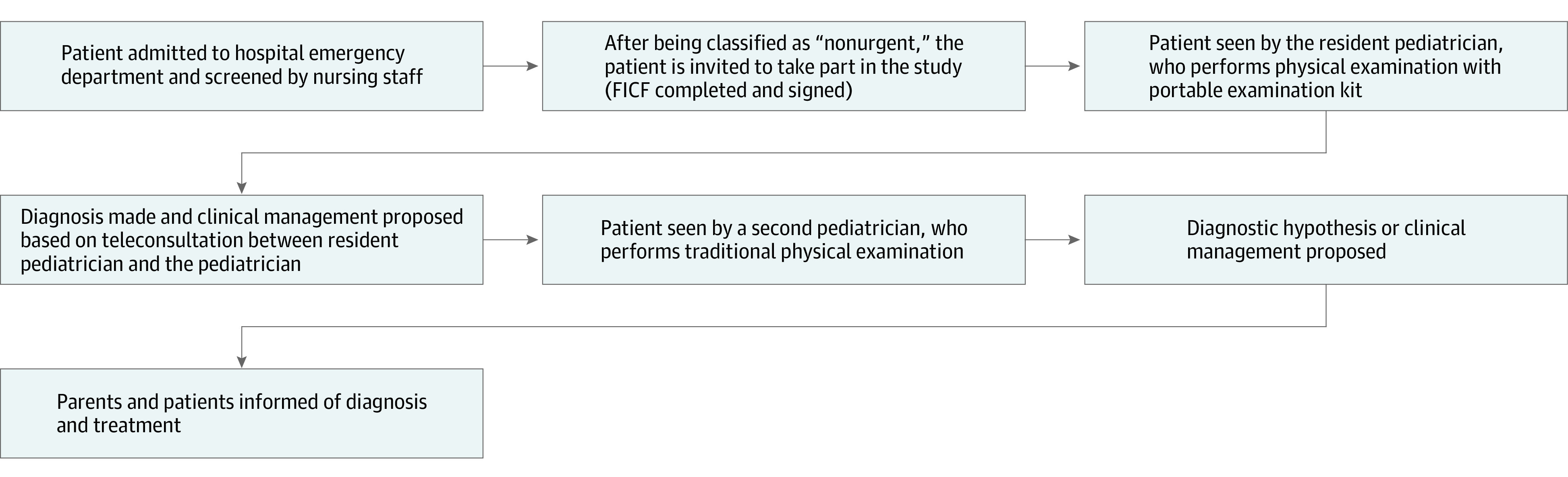
Flowchart of Patient Consultations in the Study FICF indicates free informed consent form.

### Statistical Analysis

Continuous variables were expressed as means with SDs or medians with IQRs according to the results of the Kolmogorov-Smirnov test for normality. Categorical variables were expressed as absolute and relative frequencies.

The primary outcome was concordance between measurements from the mobile device vs measurements from the in-person clinical assessment.^18^ The McNemar test was used to check for equal proportions of positive results in the tests, and the κ coefficient with 95% CIs was used to test the reproducibility of the results. The Landis and Koch concordance criteria were used to interpret the results of the κ test.^19^

The secondary outcome was the sensitivity and specificity of the mobile medical device. Sensitivity and specificity were calculated for each variable considering the results of the conventional consultation as the gold standard.

A 2-sided *P* = .05 was the significant threshold for all tests. All analyses were performed using IBM SPSS Statistics software, version 20.0 (IBM Corporation).

## Results

The main characteristics of the patients included in the study are shown in Table 1. Among 690 patients, the median (IQR) age at study entry was 5 (1-9) years; 348 (50.4%) were female. A total of 331 patients (48.0%) presented with a chronic disease, 221 (32.0%) were using long-term medication, and 93 (13.5%) reported having an allergy. Data on patient race and ethnicity were not available because the participating hospitals did not collect this information in their systems.

**Table 1.** Characteristics of Patients Included in the Study

Characteristic	Patients, No./total No. (%) (N = 690)
Age, median (IQR), y	5 (1-9)
Sex	
Male	342/690 (49.6)
Female	348/690 (50.4)
Respiratory rate, median (IQR), breaths/min	24 (20-30)
Weight, median (IQR), kg	16 (9-32)
Type of insurance	
Public	498/690 (72.2)
Private	192/690 (27.8)
No chronic disease	359/690 (52.0)
Chronic disease^a^^,^^b^	331/690 (48.0)
Cardiac	31/331 (9.4)
Dermatological	9/331 (2.7)
Endocrinological	13/331 (3.9)
Gastrointestinal	26/331 (7.9)
Genitourinary	8/331 (2.4)
Hematological/oncological	18/331 (5.4)
Neurological/psychiatric	75/331 (22.7)
Ophthalmic	7/331 (2.1)
Kidney	35/331 (10.6)
Respiratory	82/331 (24.8)
Rheumatological	36/331 (10.9)
Other	23/331 (6.9)
Long-term medication use^c^	
No	469/690 (68.0)
Yes	221/690 (32.0)
Allergy^d^	
No	597/690 (86.5)
Yes	93/690 (13.5)
Final diagnosis^b^	
Dermatological	111/690 (16.1)
Gastrointestinal	27/690 (3.9)
Genitourinary	120/690 (17.4)
Neurological	71/690 (10.3)
Respiratory	253/690 (36.7)
Fever without a focus	108/690 (15.7)
Other	252/690 (36.5)

^a^ Chronic disease was defined as the presence of more than 1 disease or condition at the same time.

^b^ Some patients presented with 2 or more chronic diseases or diagnoses.

^c^ Long-term medication was defined as medications used for an extended period to treat chronic diseases.

^d^ Allergy was defined as any allergic process associated with drugs, food, or the environment.

When analyzing the outcome of auscultations, it should be noted that the sample for this variable was smaller than the sample for other outcomes (samples ranged from 628-640 patients depending on the variable). This smaller sample size was associated with auscultations that could not be performed successfully, particularly in infants. Significantly more auscultations in infants were adversely affected by crying and agitation when auscultation was performed using the device (250 of 254 auscultations [98.4%]; 49 heart murmur, 45 heart rhythms, 48 heart sounds, 53 lung adventitious sounds, and 55 lung vesicular sounds) than in a conventional consultation (29 of 254 auscultations [11.4%]; 5 heart murmur, 2 heart rhythms, 5 heart sounds, 7 lung adventitious sounds, and 10 lung vesicular sounds) (eTable 1 in Supplement 2).

The concordance values (primary outcome) were 90% or greater for variables associated with skin examination (94% for rash, 100% for hemorrhagic suffusion, and 95% for signs of secondary infection), characteristics of the mucosa (98% for hydration and 97% for coloring), and heart (95% for murmur, 97% for rhythms, and 98% for sounds), lung (91% for adventitious sounds, 97% for vesicular sounds, and 90% for fever), and abdominal (92% for abdominal sounds) auscultations. The values were lower for otoscopy (72% for the ear canal and 86% for the tympanic membrane), throat and oral examination (72%), and rhinoscopy (79% for mucosa and 81% for secretion) (Table 2). Reliability as measured by the κ coefficient was fair to moderate. For most of the variables, the proportions of the results between groups according to the McNemar test were similar.

**Table 2.** Concordance Between Measurements From Physical Examination Using a Telemedicine Mobile Medical Device vs Conventional In-Person Physical Examination

Variable	Patients, No.	Measurement concordance, %	Reproducibility			*P* value for McNemar test for equal proportions of positive test results
			κ Coefficient (95% CI)	*P* value^a^	κ Concordance	
Skin						
Rash	690	94	0.55 (0.43 to 0.66)	<.001	Moderate	>.99
Hemorrhagic suffusion	690	100	0.83 (0.54 to 1.00)	<.001	Perfect	.50
Signs of secondary infection	690	95	0.55 (0.41 to 0.68)	<.001	Moderate	.61
Otoscopy						
Ear canal	690	72	0.35 (0.27 to 0.42)	<.001	Fair	<.001
Tympanic membrane	518^b^	86	0.44 (0.32 to 0.54)	<.001	Moderate	.01
Throat and oral examination	688	72	0.29 (0.21 to 0.37)	<.001	Fair	.02
Heart auscultation						
Murmur	635	95	0.39 (0.21 to 0.55)	<.001	Fair	>.99
Rhythms	640	97	0.24 (−0.01 to 0.47)	<.001	Fair	.09
Sounds	635	98	0.24 (0.01 to 0.53)	<.001	Fair	.39
Lung auscultation						
Adventitious sounds	628	91	0.33 (0.18 to 0.46)	<.001	Fair	.89
Vesicular sounds	630	97	0.07 (−0.02 to 0.26)	.08	Slight	.66
Fever	648	90	0.32 (0.18 to 0.45)	<.001	Fair	<.001
Rhinoscopy						
Mucosa	681	79	0.29 (0.20 to 0.37)	<.001	Fair	<.001
Secretion	681	81	0.37 (0.22 to 0.46)	<.001	Fair	.01
Mucosa						
Hydration	681	98	0.43 (0.19 to 0.64)	<.001	Moderate	>.99
Coloring	681	97	0.26 (0.06 to 0.47)	<.001	Fair	.38
Abdominal sounds	687	92	0.06 (−0.04 to 0.18)	.06	Slight	.21

^a^ Significant *P* values (significance threshold of *P* = .05) indicate that the κ value differed from 0.

^b^ The sample was smaller because of the difficulty observed in visualizing the tympanic membrane during remote physical examination (n = 130) and traditional in-person physical examination (n = 71).

The specificity and sensitivity for the mobile medical device (considering the results of the conventional consultation as the gold standard) are shown in Table 3. The specificity of the test was greater than 70% (varying from 74.5% for the ear canal to 99.7% for hemorrhagic suffusion) for all of the variables analyzed. The sensitivity was greater than 52% for skin examination (58.0% for rash and 54.8% for signs of secondary infection), throat and oral examination (52.7%), and otoscopy (66.1% for the ear canal and 64.4% for the tympanic membrane). The lowest sensitivity was found for abdominal sounds (8.8%). When a sensitivity analysis was performed including only patients with a diagnosis associated with a skin condition, there were improvements in sensitivity and concordance (eTable 2 in Supplement 2).

**Table 3.** Sensitivity and Specificity of the Mobile Medical Device

Variable	Patients, No.	Sensitivity (95% CI), %	Specificity (95% CI), %
Skin examination			
Rash	690	58.0 (47.7-67.8)	96.7 (95.0-98.0)
Hemorrhagic suffusion	690	100 (47.8-100)	99.7 (98.9-100)
Signs of secondary infection	690	54.8 (38.7-73.2)	97.7 (96.2-98.7)
Otoscopy			
Ear canal	690	66.1 (58.3-73.2)	74.5 (70.5-78.2)
Tympanic membrane	518	64.4 (50.9-76.4)	89.1 (85.9-91.8)
Throat and oral examination	688	52.7 (44.8-60.5)	78.5 (74.7-82.0)
Auscultation			
Heart			
Murmur	635	41.4 (23.5-61.1)	97.4 (95.7-98.5)
Rhythms	640	37.5 (8.5-75.5)	97.9 (96.5-98.9)
Sounds	635	33.3 (4.3-77.7)	98.7 (97.5-99.4)
Lung			
Adventitious sounds	628	37.0 (23.2-52.5)	95.0 (92.9-96.6)
Vesicular sounds	630	7.7 (2.0-36.0)	98.5 (97.2-99.3)
Other			
Fever	648	51.4 (34.0-68.6)	92.6 (90.3-94.6)
Rhinoscopy secretion	681	43.1 (34.6-51.8)	91.0 (88.3-93.3)
Mucosa			
Hydration	681	42.9 (17.7-71.1)	98.9 (97.8-99.6)
Coloring	681	23.5 (6.8-49.9)	98.8 (97.6-99.5)
Abdominal sounds	687	8.8 (1.9-23.7)	96.8 (95.1-98.0)

## Discussion

This nonrandomized controlled trial yielded satisfactory results for the medical device evaluated when the device was compared with a conventional consultation for the outcomes of measurement concordance and accuracy. The results suggested that the device could be used for reliable otoscopy (otoscope), heart and lung auscultation (digital stethoscope), skin examination (integrated camera), body temperature measurement (integrated thermometer), and throat and oral examination (tongue depressor). Our findings for otoscopy outcomes were consistent with values reported previously for other digital devices that provided high-quality visualization^3,20,21^ and with other studies using the same device.^5,9^ The concordance values in our study were similar to those reported by McConnochie et al,^4^ who found a concordance of 89% between teleconsultations and conventional consultations, and by Rappaport et al^21^ and Shah et al,^22^ who observed moderate concordance (κ = 0.503) between diagnoses using conventional otoscopy vs a digital device (CellScope Oto; CellScope, University of California Berkeley). In addition, some studies^7,18^ found that an electronic stethoscope was preferred over a conventional stethoscope, and the concordance (measured by the κ coefficient) between measurements from telemedicine and in-person examination in those studies was similar to the concordance found in our study.

For the outcome of device accuracy, the values found in the current study were similar to those obtained by Zenk et al^23^ for remote auscultation with an electronic stethoscope (sensitivity of 77% for correct identification of patients with an irregular cardiac rhythm and 86% for correct identification of patients with a regular cardiac rhythm). In a study by Kleinman et al,^24^ accuracy was better with a digital otoscope than a conventional one. Previous studies^5,9^ have reported the ability of mobile medical devices to provide a more complete view of the ear canal and tympanic membrane.

Electronic and digital stethoscopes were designed to improve auscultation by amplifying sound.^5,7^ Although the device used in this study provided good results in terms of measurement concordance and accuracy compared with a conventional stethoscope, there was a higher percentage of auscultations that could not be performed satisfactorily in infants, possibly because the greater amplification of sounds from the environment or the patient (ie, crying and agitation) interfered with the quality of the auscultation. Certain variables can therefore make it difficult to perform auscultations. These variables, such as age group, may be related to the patients themselves. According to Pasterkamp and Zielinski,^25,26^ thoracic auscultation represents a major challenge during a pediatric physical examination. Our results suggested that most of the heart and lung auscultations that could not be performed satisfactorily occurred in infants rather than noninfants; these findings were corroborated by previous studies,^27,28,29^ which reported that heart sounds during auscultation were adversely affected in more agitated and active children as well as in infants, regardless of the physician’s experience. Despite the technological advances that have occurred in the last 25 years, the findings of the present study confirmed the challenge that thoracic auscultation in patients with this profile still poses.

A study by Dumas et al^30^ evaluated abdominal auscultation in 8 infants using an electronic stethoscope. The authors found that environmental and other types of sounds adversely affected auscultation and at times even prevented an adequate assessment.^30^ In the present study, the poor sensitivity observed for abdominal sounds (8.8%) may suggest that there were a large number of false-negative results. According to Baid,^31^ there is wide variation in the techniques adopted by physicians during an abdominal auscultation as well as differences in the duration of the auscultation and the region examined, potentially making the assessment highly subjective. All of these factors can have implications for the results of examinations for the same patient when examined by different physicians.

It should be noted that this modality of telemedicine is indicated for patients with stable, nonurgent conditions, despite the complexity of the underlying disease. The future use of mobile medical devices may be most beneficial in 2 main settings: allowing access to health care in distant and vulnerable locations and guaranteeing safe hospital discharge with associated home telemonitoring for patients with conditions of different complexity (eg, postoperative patients or patients receiving bone marrow transplants). Furthermore, the use of a mobile medical device may reduce costs by enabling improvements in the allocation of hospital resources, prevention and earlier diagnosis of complications among discharged patients, reduction in exposure to preventable diseases among vulnerable patients, and expansion of access to qualified care in the primary care context.

### Limitations

This study has several limitations. The main limitation is that the comparator (in-person physical examination) also has certain limitations regarding subjectivity, compromising the results of the teleinterconsultation using a mobile medical device. Furthermore, this study includes patients in different age groups with different potential diagnoses, making it difficult to assess the accuracy of the device when used with specific populations (such as patients with heart disease). However, future stages of this study will include assessment of the device among subgroups of patients with chronic diseases and analyses of cost-effectiveness, considering that the incorporation of this technology requires an investment of $18 000 Brazilian reals (R$; $3496 US dollars based on the mean 2022 exchange rate of R$1.00 = US $ 0.1942) for the device and R$769 (US $149) per month for the platform.

## Conclusions

This nonrandomized controlled trial found that remote physical examination using a mobile device among patients who had stable conditions with or without chronic diseases had satisfactory measurement concordance with in-person physical examination for some variables, such as heart and lung auscultation, otoscopy, throat and oral examination, and skin examination. The concordance was limited for heart and lung auscultation in infants and abdominal auscultation in children of all ages. Measurements from remote physical examination using the mobile medical device were comparable with those from in-person examination in children older than 2 years. These findings suggest that the device may be a useful tool for examining patients who do not have access to a health care center. Telemedicine may therefore be an alternative to in-person examination in certain contexts, optimizing access to health care services and reducing social and geographic barriers.

## Notes

Supplement 1.
Trial Protocol and Statistical Analysis Plan

Supplement 2. **eFigure.** CONSORT Flowchart for a Single-Arm Nonrandomized Study of Pediatric Patients **eTable 1.** Number of Adversely Affected Auscultations in Infants and Noninfants **eTable 2.** Sensitivity Analysis Based on Patients With a Diagnosis Associated With a Skin Condition

Supplement 3.
Data Sharing Statement
