# Supplementary material for: Assessment of Pediatric Telemedicine Using Remote Physical Examinations With a Mobile Medical Device: A Nonrandomized Controlled Trial
Source: JAMA Netw Open. 2023 Feb 2;6(2):e2252570. doi: 10.1001/jamanetworkopen.2022.52570 (PMC9896296; doi:10.1001/jamanetworkopen.2022.52570)
Supplement: Supplement 1. — Trial Protocol and Statistical Analysis Plan [file jamanetwopen-e2252570-s001.pdf]

SUPPLEMENTARY MATERIAL

TRIAL PROTOCOL AND STATISTICAL ANALYSIS PLAN SUBMITTED TO THE IRB/ETHICS COMMITTEE (ENGLISH - TRANSLATE)

TITLE: Assessment of pediatric telemedicine using a mobile medical device: A nonrandomized controlled trial

SUBTITLE: Assessment of telemedicine by means of tele-interconsultation in pediatric care using a mobile medical device: A nonrandomized controlled trial

This document presents a translated copy of the TRIAL PROTOCOL AND STATISTICAL ANALYSIS PLAN that was submitted to the Committee for Ethics in Research at the Pequeno Príncipe Hospital (CAAE ref. no.: 15828819·8·1001·0097) and the Committee for Ethics in Research at the José Luiz Egydio Setubal Foundation (CAAE ref. no.: 15828819·8·3001·5567).

METHODOLOGY

This study is a prospective, single-arm, non-randomized clinical trial to compare a mobile physical device linked to telemedicine with traditional physical examination in the care of patients in the emergency department of a pediatric hospital.

RESEARCH LOCATION

This study was performed in two pediatric, tertiary care and high complexity hospital (Pequeno Príncipe Hospital and Sabará Hospital), located in Curitiba and São Paulo, respectively.

STUDY DESIGN

This study will perform in two steps. The first is the integration of the Tytocare® system with the hospital's electronic system, to enable the operationalization of telemedicine technology. This step will not include patients and will conduct an estimated time of two to four months to complete. The second step refers to patient care through telemedicine, using the Tytocare® medical device

and the teleinterconsultation modality, that is, from doctor to doctor. This step will include 698 patients with Informed Consent Form and with an estimated time of ten months.

1. Integration of the Tytocare operating system with the electronic system for telemedicine feasibility

This step will not include patient participation. This step will allow the data and images generated by Tytocare® to be send to the hospital's electronic system. The data captured will be transmitted to the devices and, through the Tytocare® system, will be stored in a database hosted in a "cloud", where the security and integrity of the data will be guaranteed by Tytocare®. Once stored in the Tytocare® system, it will be necessary to develop a system to allow interoperability between the Tytocare® system and the Hospital's electronic system. Through the Web Service layer, data will be retrieved from the Tytocare system database by the electronic system, and insert into the patient's medical record as the care steps begin, following safety rules access and persistence of the system.

It is important to highlight that data security and integrity will be guaranteed at four different times: a) on the capture device; b) in the traffic from that to the Tytocare system; c) from this to the patient's medical record system; d) and in the medical record system.

2. Patient care using Tytocare® in the emergency room through teleinterconsultation

The objectives of this step will be to enable teleinterconsultation between physicians, using Tytocare®, as well as to compare the data from the physical examination obtained by Tytocare® versus the data from the physical examination performed in a traditional way, by the physician in a routine consultation, as diagnostic support.

PARTICIPANTS

Eligible participants in this study will be those classified as low risk (classified as green) in the triage performed by nurses in the emergency room.

Both hospitals have a risk classification protocol to stratify and classify all patients who arrive at the Emergency room. This classification is performed by the nurse and the protocol consists of five color codes (red, orange, yellow, blue and green):

- Red: serious patient, considered as an emergency, with immediate care;
- Orange: patient considered urgent, with care within a maximum period of 15 minutes;
- Yellow: patients who do not present conditions that characterize medical urgency, however, are identified as patients who require particular conditions for prioritization. As those who have comorbidities, associated with clinical conditions with potential risk for complications, with a goal of care of up to one hour;
- Green: patients who do not present clinical conditions that characterize medical urgency, with an average time of care within four hours;
- Blue: patients who have specific needs for procedures. These patients will not be classified because they do not have clinical circumstances, but they will be prioritized over those classified as non-urgent (Green), such as: tube replacement, administration of medication or dressings. Your average handle time target is two hours.

ELEGIBILITY CRITERIA

Inclusion criteria:

Will be eligible:

- Patients from 0 to 18 years old;
- Patients of both sexes;
- Patient treated in the Emergency Service of the public and private system;
- Patients classified in the green code (low risk) in the screening perform in Emergency Care;
- Patients in which one of the parents or legal guardian consents and signs the Informed Consent Term.

Exclusion criteria:

- Patients classified in red, orange, yellow and blue codes;

- Patients in which the legal guardian or one of the parents does not consent to participation;
- Patient who does not agree to sign the Term of Assent/consent.

FLOWCHART OF THIS STUDY

The single-arm, non-randomized clinical trial in second step will consist of selecting patients classified as code green (low risk), who will undergo two medical consultations and two physical examinations (Tytocare® and traditional), with the aim of comparing the results between these two modalities of examination and diagnosis. The flowchart will be as detailed in the steps below and illustrated in figure 1.

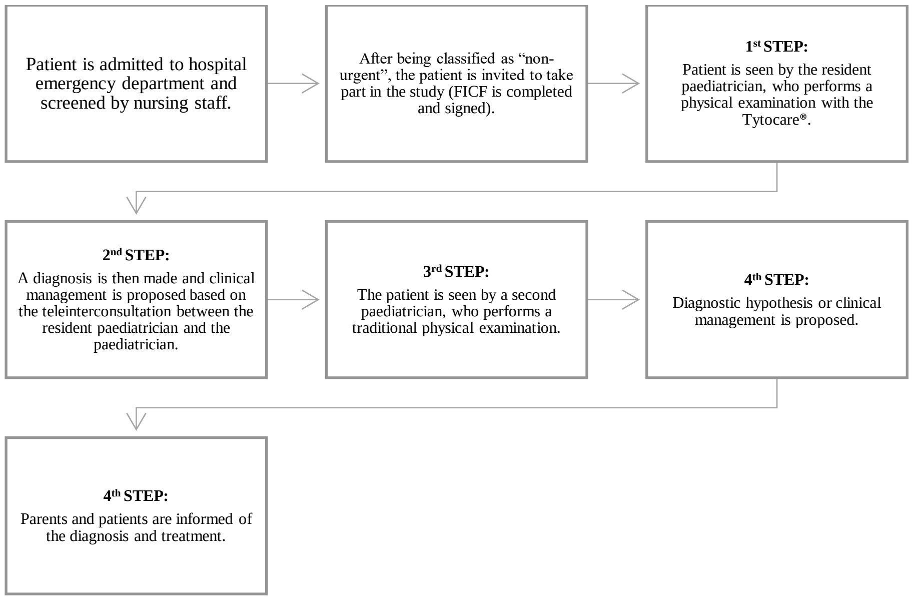

Figure 1. Flowchart of patient consultations in the study.

Step 1: The patient, after going through the nursing screening and being classified as code green and accepting to participate in the study, will be submitted to the first consultation with Tytocare® intervention, in the room 1. This consultation will be performed by a pediatric resident of the first year, whose inexperience should mimic that of the non-specialist doctor in the health units. The resident will collect information related to past medical history, current symptoms, and will perform a physical examination using Tytocare®. This device allows obtaining the following finding: heart rate, respiratory rate, body temperature, cardiac, pulmonary and abdominal

auscultation, otoscopy and oroscopy. In this room, Tytocare® and an attached computer will be available for data recording by the Pediatrics Resident.

Step 2: As the data is entered into the computer, it will be transmitted from the Tytocare® platform to the eletronic system, and a second physician, called PEDIATRICIAN 1, will receive the results and images of the physical examination obtained by Tytocare®. PEDIATRICIAN 1 will be allocated in a room, called the situation room, guiding the resident and confirming the patient's care, thus configuring a second opinion on the case, thus characterizing the teleinterconsultation. These two doctors will decide together at a diagnostic hypothesis, diagnosis and/or course to be taken, which will be recorded in the data collection form (electronic document in the electronic system).

Step 3: The patient will be invited to go to the second consultation, which we will call a traditional consultation, and will be performed by another pediatrician, called PEDIATRICIAN 2. In this consultation, the doctor will perform the entire routine procedure (anamnesis with physical examination), without the use of Tytocare®. The traditional physical examination consists of obtaining the same clinical parameters using usual means or equipment such as a digital thermometer, sphygmomanometer, stethoscope and otoscope. This second consultation will take place in the hospital's emergency medical office, according to the service's demand flow. This physician will record your finding (vital signs, diagnostic hypothesis and conducts) on a patient registration form in the electronic system. In this moment, PEDIATRICIAN 2 will not have access to information registered for PEDIATRICIAN 1, in order to not compromise the results.

Step 4: The PEDIATRICIAN 2 will decide and communicate the diagnosis to the parents. Your conduct and/or diagnosis and treatment proposal will perform.

If there is a divergence between diagnoses, this will be observed in real time by the study coordinator and, in order to ensure patient safety, PEDIATRICIAN 2 can consult and discuss with the resident and PEDIATRICIAN 1. Furthermore, the PEDIATRICIAN 1 can use the results and images from the Tytocare® physical examination, however, without being able to make changes to the information in the data collection document.

SAMPLE SIZE AND STATISTICAL ANALYSIS

When comparing physical examinations by the two methods (captured by telemedicine and traditional physical examination), two statistical elements are involved, the comparison of paired proportions and the agreement between methods.

Comparison of paired proportions

This type of comparison is usually done using the McNemmar test, as it considers the pairing of samples when comparing proportions. The sample calculation is then given considering that records from group A are paired with records from group B, this occurs in cases where two methods evaluate the same samples. McNemmar's test also considers that one wants to verify the equality of probability of a finding.

The sample size calculation requires some parameters. For our calculation, we consider four parameters. We will use the alpha of 0.05 and the statistical power (Bheta) of 0.80.

The expected total proportions of change from positive to negative and from negative to positive are also considered. For the calculation of the order of these two proportions it does not matter. We see in the following table a combination of proportions that result in different sample sizes.

**Prop 1\Prop 2**	**0.10**	**0.15**	**0.20**	**0.25**
**0.05**	**468**	155	85	57
**0.10**		781	233	120
**0.15**			1095	311
**0.20**				1408

If these two proportions were too large, it would indicate that the methods are not comparable, therefore, and also for the practical aspect of the study, a sample size of 468 was chosen for the aspect of comparing paired proportions.

Concordance between methods

Verification of concordance between methods will be performed using the kappa statistical method. Again we have a combination of parameters in the calculation to establish the sample size.

Bujang's proposal (2017) uses the quantities of categories, power and alpha for the sample calculation. Assuming 80% power with an alpha of 0.05 and knowing that we have two possible categories in each response (found or not found), we have the following combination of suggested sample sizes for each combination of proportions.

**Prop 1 \ Prop 2**	**0.0**	**0.3**	**0.5**	**0.7**
**0.2**	194			
**0.3**	85			
**0.4**	47	**698**		
**0.5**	29	169		
**0.6**	20	72	**563**	
**0.7**	14	39	133	
**0.8**	10	23	54	363
**0.9**	7	14	27	79

Considering a statistical power of 90%, we have the following suggestion of sample sizes.

**Prop 1 \ Prop 2**	**0.0**	**0.3**	**0.5**	**0.7**
**0.2**	259			
**0.3**	113			
**0.4**	62	**927**		
**0.5**	38	222		
**0.6**	25	94	**742**	
**0.7**	17	49	171	
**0.8**	12	28	68	471
**0.9**	8	17	32	96

With this information, this method suggests a sample size of 698 patients (according to the proportions of the study, using the 80% power table and considering 0.3 and 0.4 proportions).

Conclusion about the sample size

Thus, considering the two methods, a sample size of around 698 patients is suggested of this study.

DATA COLLECT

Data collection will perform through an electronic document in the electronic system at the study hospital, specially prepared to collect and record research data. This document will present the following information about patient: general data (age, weight, comorbidities), clinical history, physical examination (heart rate, respiratory rate, temperature, blood pressure, cardiac, pulmonary and abdominal auscultation, otoscopy and oroscopy), diagnosis, diagnostic hypothesis and management.

The data collection instrument will be the same for the consultation with Tytocare and the traditional consultation. However, PEDIATRICIAN 2 will not have access to the PEDIATRICIAN 1 results registration form and vice versa, to ensure data confidentiality and research suitability.

A study coordinator will be able to access the two records, in order to see if there is a discrepancy between the diagnoses or diagnostic hypotheses. When this occurs, the coordinator will inform PEDIATRICIAN 2 if he wants to discuss the case with PEDIATRICIAN 1 and the pediatric resident who treated the patient with Tytocare®.

ANALYSIS OF RESULTS

The results of physical examinations (Tytocare® vs traditional) and diagnostic hypotheses will be analyzed by comparing paired proportions and agreement between methods.

After meeting the expected sample, we will analyze whether the statistical tests will demonstrate the hypothesis of the study, that the physical examination obtained by Tytocare or diagnosis supported by the device is equivalent to the traditional physical examination, in the parameters of clinical signs and diagnostic hypothesis, as well as that Tytocare can be delivered to a telemedicine service.
