# Supplementary material for: Assessment of Pediatric Telemedicine Using Remote Physical Examinations With a Mobile Medical Device: A Nonrandomized Controlled Trial
Source: JAMA Netw Open. 2023 Feb 2;6(2):e2252570. doi: 10.1001/jamanetworkopen.2022.52570 (PMC9896296; doi:10.1001/jamanetworkopen.2022.52570)
Supplement: Supplement 2. — eFigure. CONSORT Flowchart for a Single-Arm Nonrandomized Study of Pediatric Patients eTable 1. Number of Adversely Affected Auscultations in Infants and Noninfants eTable 2. Sensitivity Analysis Based on Patients With a Diagnosis Associated With a Skin Condition [file jamanetwopen-e2252570-s002.pdf]

Supplemental Online Content

Wagner R, Lima TC, Silva MRT, et al. Assessment of pediatric telemedicine using remote physical examinations with a mobile medical device: a nonrandomized controlled trial. *JAMA Netw Open*. 2023;6(1):e2252570. doi:10.1001/jamanetworkopen.2022.52570

eFigure. CONSORT Flowchart for a Single-Arm Nonrandomized Study of Pediatric Patients

eTable 1. Number of Adversely Affected Auscultations in Infants and Noninfants

eTable 2. Sensitivity Analysis Based on Patients With a Diagnosis Associated With a Skin Condition

This supplemental material has been provided by the authors to give readers additional information about their work.

© 2023 Wagner R et al. *JAMA Network Open*.

eFigure. CONSORT Flowchart for a Single-Arm Nonrandomized Study of Pediatric Patients

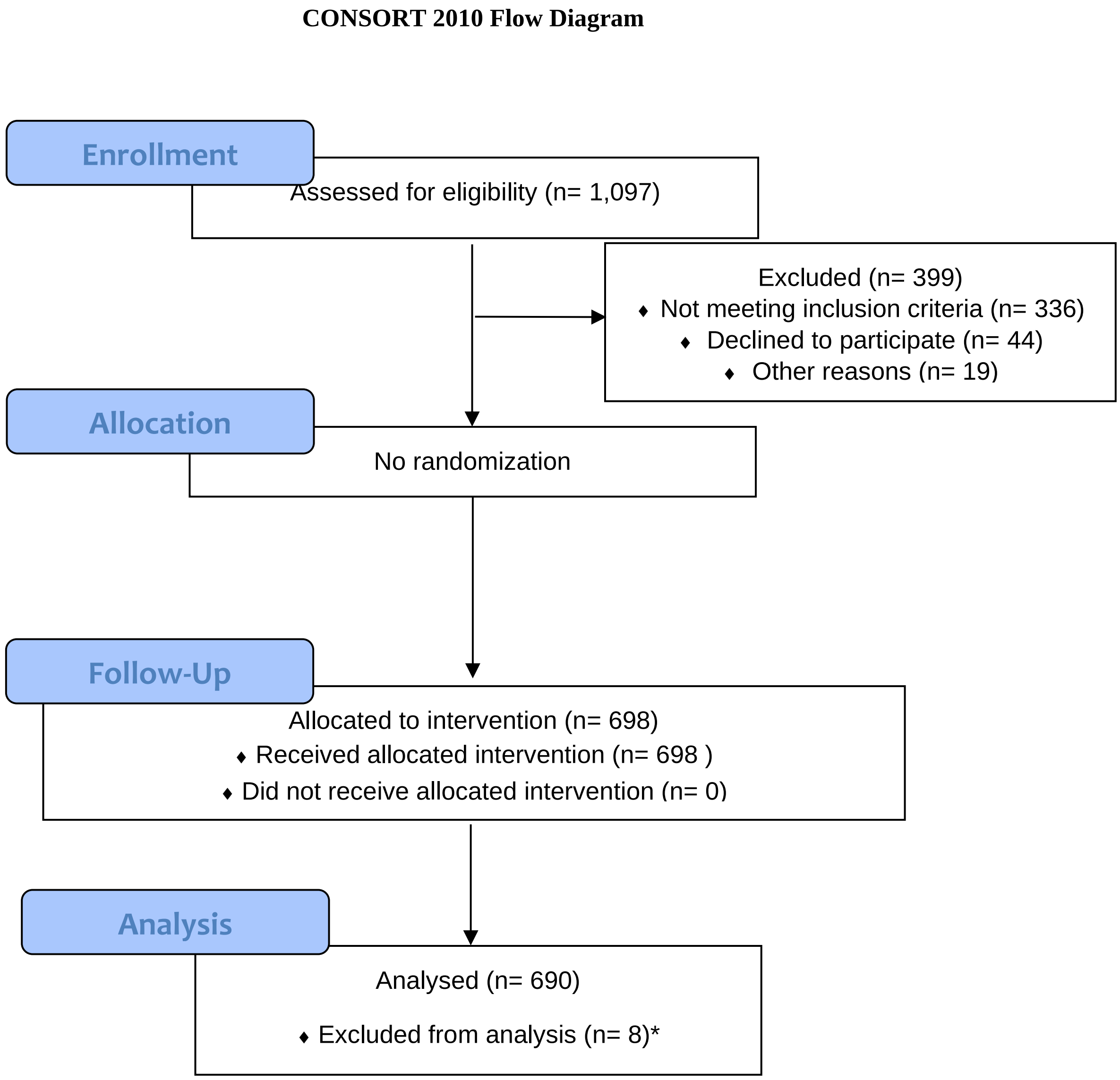

***Note: 8 patients were excluded due to uncollected data.**

© 2023 Wagner R et al. *JAMA Network Open*.

eTable 1. Number of Adversely Affected Auscultations in Infants and Noninfants

	**Heart auscultation - murmur**	**Heart auscultation - rhythms**	**Heart auscultation - sounds**	**Lung auscultation - adventitious sounds**	**Lung auscultation - vesicular sounds**
**Infant (n=254)**					
**Tyto®**	49 (19%)	45 (17%)	48 (19%)	53 (21%)	55 (22%)
**Conventional consultation**	5 (2%)	2 (1%)	5 (2%)	7 (3%)	10 (4%)
**Ineligible**	**51 (20%)**	**45 (18%)**	**49 (19%)**	**54 (21%)**	**57 (22%)**
**Non-infant (n=436)**					
**Tyto**	4 (0·9%)	4 (0·9%)	5 (1·2%)	5 (1·2%)	5 (1·2%)
**Conventional consultation**	0 (0%)	1 (0·2%)	1 (0·2%)	1 (0·2%)	1 (0·2%)
**Ineligible**	4 (0·9%)	5 (1·1%)	6 (1·4%)	6 (1·4%)	6 (1·4%)

© 2023 Wagner R et al. *JAMA Network Open*.

eTable 2. Sensitivity Analysis Based on Patients With a Diagnosis Associated With a Skin Condition

Variable	n	Sensitivity	Specificity	Area	Standard error	p value	Kappa analysis	McNemar	Concordance
Skin - rash	127	67·6 (50·2-82·0)	87·8 (79·2-93·7)	0·78 (0·68-0·88)	0·05	<0·001	Moderate	1·00	82%
Skin - haemorrhagic suffusions	127	100·0 (29·2-100·0)	100·0 (97·1-100·0)	1·00 (1·00-1·00)	0·000	0·003	Perfect agreement	1·00	100%
Skin - signs of secondary infection	127	62·5 (42·7-78·9)	89·5 (81·5-94·8)	0·76 (0·65-0·87)	0·055	<0·001	Moderate	0·83	83%

© 2023 Wagner R et al. *JAMA Network Open*.
