# Supplementary material for: Assessment of Pediatric Telemedicine Using Remote Physical Examinations With a Mobile Medical Device: A Nonrandomized Controlled Trial
Source: JAMA Netw Open. 2023 Feb 2;6(2):e2252570. doi: 10.1001/jamanetworkopen.2022.52570 (PMC9896296; doi:10.1001/jamanetworkopen.2022.52570)
Supplement: Supplement 3. — Data Sharing Statement [file jamanetwopen-e2252570-s003.pdf]

# Data Sharing Statement

Wagner. Assessment of Pediatric Telemedicine Using Remote Physical Examinations With a Mobile Medical Device. *JAMA Netw Open*. Published February 02, 2023. doi:10.1001/jamanetworkopen.2022.52570

**Data**
**Data available:** No
